# The impact of dose modification and temporary interruption of ibrutinib on outcomes of chronic lymphocytic leukemia patients in routine clinical practice

**DOI:** 10.1002/cam4.2998

**Published:** 2020-03-18

**Authors:** Sameer A. Parikh, Sara J. Achenbach, Timothy G. Call, Kari G. Rabe, Wei Ding, Jose F. Leis, Saad S. Kenderian, Asher A. Chanan‐Khan, Amber B. Koehler, Susan M. Schwager, Eli Muchtar, Amie L. Fonder, Kristen B. McCullough, Adrienne N. Nedved, Matthew D. Smith, Susan L. Slager, Neil E. Kay, Heidi D. Finnes, Tait D. Shanafelt

**Affiliations:** ^1^ Division of Hematology Department of Medicine Mayo Clinic Rochester MN USA; ^2^ Division of Biomedical Statistics & Informatics Mayo Clinic Rochester MN USA; ^3^ Department of Hematology and Oncology Mayo Clinic Phoenix AZ USA; ^4^ Division of Hematology and Oncology Mayo Clinic Jacksonville FL USA; ^5^ Department of Pharmacy Mayo Clinic Rochester MN USA; ^6^ Division of Hematology Stanford University School of Medicine Palo Alto CA USA

## Abstract

To study the impact of dose modification and temporary interruption of ibrutinib in routine clinical practice, we conducted a retrospective study of consecutive CLL patients treated with ibrutinib outside the context of a clinical trial at Mayo Clinic, (Rochester, MN) from 11/2013 to 12/2017. Of 209 patients, 131 (74%) had unmutated *IGHV*, 38 (20%) had *TP53* disruption, and 47 (22%) were previously untreated. A total of 87/209 (42%) patients started reduced dose ibrutinib (<420 mg daily; n = 43, physician preference; n = 33, concomitant medications; and n = 11, other). During 281 person‐years of treatment, 91/209 patients had temporary dose interruption (54%, nonhematologic toxicity; 29%, surgical procedures; 10%, hematologic toxicity; and 7%, other). After a median follow‐up of 24 months, the estimated median event‐free survival (EFS) was 36 months, and median overall survival (OS) was not reached. On multivariable analyses, temporary ibrutinib interruption (hazard ratio [HR]: 2.37, *P* = .006) and *TP53* disruption at ibrutinib initiation (HR: 1.81, *P* = .048) were associated with shorter EFS, whereas only *TP53* disruption (HR: 2.38, *P* = .015) was associated with shorter OS. Initial ibrutinib dose and dose modification during therapy did not appear to impact EFS or OS. These findings illustrate the challenges associated with continuous oral therapy with ibrutinib in patients with CLL.

## Graphical abstract

In this single‐institution study of CLL patients receiving ibrutinib therapy outside the context of a clinical trial, approximately 40% patients initiated ibrutinib at a reduced dose (<420 mg daily, primarily due to concomitant medications that may increase ibrutinib toxicity and physician prescribing patterns). In addition, approximately 50% patients subsequently undergo a dose modification and/or a dose interruption after initiation of ibrutinib therapy. Finally, although ibrutinib starting dose and dose modifications did not impact event free survival and overall survival, temporary dose interruptions during therapy were associated with shorter event free survival and shorter overall survival.

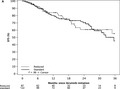

## 1 INTRODUCTION

Multiple clinical trials have demonstrated the safety and efficacy of ibrutinib, a first‐in‐class Bruton's tyrosine kinase (BTK) inhibitor, in the treatment of patients with chronic lymphocytic leukemia (CLL).^1^, ^2^, ^3^ These data led to the approval of ibrutinib for patients with previously untreated and relapsed/refractory CLL by both the Food and Drug Administration (FDA) and the European Medicines Agency (EMA). Long term follow‐up of the 132 CLL patients on single‐agent ibrutinib in the PCYC‐1102/1103 study, found the estimated 5‐year progression free survival (PFS) was 92% for treatment naïve patients and 44% for patients with relapsed/refractory CLL.^4^ A *post hoc* analysis of the RESONATE trial (a phase 3 study comparing ibrutinib to ofatumumab in relapsed/refractory CLL) found that higher treatment adherence, measured by the overall ibrutinib dose intensity in the first 8 weeks of therapy, was associated with longer PFS relative to patients with lower ibrutinib dose intensity.^5^

While the efficacy of ibrutinib in patients participating in well‐designed prospective clinical trials is encouraging, it is likely that the toxicity profile, adherence, and rates of discontinuation for reasons other than progression may differ in routine clinical practice for several reasons. First, in clinical trials, patients receive ibrutinib free of charge such that out of pocket cost considerations are not a factor in adherence. Second, patients in trials tend to be and are more likely to adhere to the prescribed treatment regimen. Third, patients in trials are typically younger, have fewer co‐morbidities and better performance status than patients treated in routine clinical practice. These considerations have important implications for the management of patients with CLL, who are typically elderly, have co‐morbid health conditions, and may live on a fixed income. Preliminary data from our group indicate that approximately two‐thirds of “real‐world” CLL patients initiating ibrutinib therapy are on concomitant medications that could increase ibrutinib levels (such as CYP3A inhibitors) and ~ 3% are on drugs that could decrease ibrutinib efficacy (such as CYP3A inducers).^6^ Mato et al recently reported that among 621 CLL patients who received ibrutinib therapy, 42% patients discontinued treatment after a median follow‐up of 17 months.^7^ Although the starting dose of ibrutinib (standard 420 mg daily vs <420 mg daily) was not associated with adverse clinical outcome in that study, the reasons for initiating lower dose ibrutinib, the proportion of patients who reduced the dose or temporarily held ibrutinib during the course of treatment, and the potential impact of such events on clinical outcome are not described. It is important to gain even more knowledge in this area since poor compliance with therapy; improper interruptions or decrease in the dose of ibrutinib may increase the risk of drug resistance and may offset the impressive response duration and survival noted with ibrutinib in clinical trials.

Using the Mayo Clinic CLL Database, we conducted a retrospective analysis to determine the reasons for ibrutinib dose modifications as well as temporary interruptions in therapy and correlated these events with outcomes in a cohort of CLL patients treated outside the context of a clinical trial.

## 2 METHODS

The Mayo Clinic CLL Database, established in 1995, includes patients with a clonal B‐cell population of the CLL immunophenotype who are seen at Mayo Clinic, Rochester, MN and who allow their medical records be used for research purposes.^8^, ^9^, ^10^, ^11^ We used this database to identify all CLL patients who received therapy with ibrutinib outside the context of a clinical trial (ie those who received commercial supply of ibrutinib). Patients were excluded from analysis if (a) they received ibrutinib therapy on a clinical trial or (b) their first treatment with ibrutinib occurred outside of Anonymous. Baseline clinical characteristics including age, sex, Rai stage, beta‐2 microglobulin, lactate dehydrogenase (LDH), immunoglobulin heavy chain gene mutation status [*IGHV*], genetic abnormalities detectable by fluorescent in situ hybridization [FISH]) and prior CLL therapy were ascertained at the time ibrutinib was initiated. *TP53* mutation assay was also performed using Sanger sequencing to detect the presence of somatic mutations involving exons 4‐9 and associated splice junctions (sensitivity of the assay is ~20%). Patients were followed until death or loss to follow‐up. Data were frozen for analysis on 14 December 2017. The Mayo Clinic Institutional Review Board approved this study.

Prior to ibrutinib start, all patients received a formal pharmacy consult with documentation of coexisting medications and potential interactions, along with recommendations (if indicated) to adjust the starting dose of ibrutinib based on concomitant medications according to the ibrutinib package instructions.^12^ The starting dose of ibrutinib was recorded for all patients. For those patients who initiated ibrutinib at a lower than standard dose (420 mg daily), the reasons for dose modification were recorded. Patients who had a dose modification or interrupted ibrutinib during the course of their treatment were also identified. The reasons for dose modification or dose interruption were recorded and categorized as follows: (a) nonhematologic toxicity (eg atrial fibrillation, bleeding, infection, myalgias, diarrhea, rash, hypertension, other); (b) hematologic toxicity; (c) drug‐drug interaction; (d) physician/patient preference; (e) procedure; (f) financial burden and (g) other. Patients who permanently discontinued therapy were identified and the reasons for stopping therapy were categorized as follows: (a) CLL progression; (b) Richter's transformation; (c) toxicity; and (d) other. Since patients in this analysis were treated as part of routine clinical practice, the frequency of follow‐up was not uniform. In general, patients were seen monthly the first 3 months after starting ibrutinib and then once every 3‐4 months while they continued active therapy. Imaging studies and/or bone marrow assessment to document responses while receiving ibrutinib therapy were performed at physician discretion. Additionally, toxicity grading was not uniformly recorded on all patients; however the package insert of ibrutinib was followed for all dose modifications and interruptions.^12^ If patients were off ibrutinib therapy for more than 60 days (for any reason), they were considered to have stopped ibrutinib permanently.

### 2.1 Statistical analysis

We used Chi‐squared tests to compare discrete variables and the Kruskal‐Wallis test to compare continuous variables. Event‐free survival (EFS) was defined as the time from initiation of ibrutinib therapy to disease progression, initiation of next line of therapy, or death due to any cause. This end‐point was chosen over progression‐free survival (PFS) given that patients in this cohort study were treated according to the treating physician's judgement, and, unlike in clinical trials, were not mandated to undergo routine scans for disease assessment at specific intervals or time‐points. Overall survival (OS) was defined as the time interval between start of therapy and the date of death (regardless of cause of death). Kaplan‐Meier plots were generated to determine EFS and OS of all patients, and to compare EFS and OS among patients who were treated with standard dose ibrutinib verses reduced dose ibrutinib. Cox proportional hazards regression analysis was used to determine factors which predicted shorter EFS and OS in the entire cohort. Time to first dose interruption was included as a time‐dependent variable. Competing risk analyses were used to analyze cause‐specific discontinuation. All statistical analyses were performed by SJA and KGR using SAS 9.4 (SAS Institute).

## 3 RESULTS

Two hundred and nine patients with CLL received therapy with ibrutinib outside the context of a clinical trial at Mayo Clinic (Rochester, MN) between November 2013 and December 2017. Of these, 162 (78%) patients received ibrutinib for relapsed/refractory CLL and 47 (22%) for progressive, treatment‐naïve CLL. The baseline characteristics of all patients are shown in Table 1. The median age at ibrutinib initiation was 69 years (range, 42‐94), and 148 (71%) were male.

**Table 1:** Baseline characteristics of all patients at the time of ibrutinib start (n = 209)

Characteristic	Total	Standard initial dose (420 mg)	Reduced initial dose (<420 mg)	*P*‐value
N	209	122	87	
Median (range) age (y)	69 (42‐94)	67 (42‐94)	72 (47‐90)	**<.001**
Males	148 (71%)	82 (67%)	66 (76%)	.18
Rai stage				
0	26 (13%)	19 (16%)	7 (9%)	.18
I‐II	62 (31%)	39 (33%)	23 (29%)	
III‐IV	110 (56%)	60 (51%)	50 (62%)	
Missing	11	4	7	
Beta‐2 microglobulin (μg/mL)				
Median (range)	4.0 (1.6‐28.2)	4.0 (1.6‐11.4)	4.7 (2.2‐28.2)	.16
Missing	113	58	55	
LDH (U/L)				
Median (range)	216 (109‐1178)	216 (117‐1178)	217 (109‐483)	.17
Missing	6	4	2	
*IGHV*				
Mutated	46 (26%)	23 (22%)	23 (31%)	.19
Unmutated	131 (74%)	80 (78%)	51 (69%)	
Missing	32	19	13	
FISH				
Standard risk (del13, +12, normal)	113 (62%)	64 (59%)	49 (65%)	.41
High risk (del11q, del17p)	70 (38%)	44 (41%)	26 (35%)	
Missing	26	14	12	
*TP53* disruption (including del17p and *TP53* mutation)				
No	151 (80%)	84 (76%)	67 (85%)	.15
Yes	38 (20%)	26 (24%)	12 (15%)	
Missing	20	12	8	
Number of prior CLL therapies				
0	47 (22%)	32 (26%)	15 (17%)	.14
1‐2	88 (42%)	45 (37%)	43 (49%)	
≥3	74 (35%)	45 (37%)	29 (33%)	

Abbreviations: FISH, fluorescent in situ hybridization; IGHV, immunoglobulin heavy chain gene mutation status; LDH: lactate dehydrogenase.

Bold indicates *P*‐value < .05

### 3.1 Baseline dose

The standard dose of ibrutinib (420 mg daily) was used as the starting dose in 122 (58%) patients. Among the remaining 87 (42%) patients, 35 patients started at 280 mg daily, 48 patients started at 140 mg daily, and 4 patients started at 140 mg orally every other day. Reasons for reduced‐dose ibrutinib at initiation included: (a) physician/patient preference (n = 43; 49%); (b) concomitant antiplatelet/anticoagulant use (n = 17; 20%); (c) concomitant therapy with CYP3A inhibitors (n = 16; 18%), and d) other (n = 11; 13%). Except for older age at ibrutinib initiation (median 72 years vs 67 years, *P* < .001), there were no significant differences in baseline characteristics among patients who received a lower starting dose of ibrutinib compared to standard dose (Table 1). When accounting for total body weight, 158 patients (76%) received ibrutinib ≥ 2.5 mg/kg/day at initiation (median [range] dose = 4.7 [2.5‐8.9] mg/kg/day) and 49 patients received < 2.5 mg/kg/day at initiation (median [range] dose = 1.8 [1.2‐2.4] mg/kg/day; baseline weight was not available for 2 patients).

### 3.2 Dose modification among those who started standard dose (420 mg daily) ibrutinib

Of 122 patients who started standard dose ibrutinib, a dose modification occurred at least once in 48 patients. Collectively, there were a total of 79 dose reductions in these 48 patients (median = 1 [range 1‐4]), and the estimated unadjusted 6‐month, 1‐year, and 2‐year cumulative incidence rate of first dose modification was 33%, 39%, and 48%, respectively. Reasons for the 79 ibrutinib dose reductions (some dose reductions were due to multiple reasons) included: (a) nonhematologic toxicity (n = 50; atrial fibrillation [n = 10], bleeding [n = 5], diarrhea [n = 5], hypertension [n = 2], infection [n = 3], musculoskeletal pain [n = 6], rash [n = 6], other [n = 18]); (b) hematologic toxicity (n = 16); (c) drug‐drug interaction (n = 9); (d) physician/patient preference (n = 5); (e) financial burden (n = 1) and (f) other (n = 4).

### 3.3 Dose modification among those who started reduced dose (<420 mg daily) ibrutinib

Of the 87 patients who initiated reduced dose ibrutinib, the dose remained unaltered during the course of treatment for 44 patients (22 patients on 280 mg daily, 20 patients on 140 mg daily and 2 patients on 140 mg every 48 hours). There were a total of 86 dose modifications in the remaining 43 patients (median = 1 [range 1‐7], estimated unadjusted 6‐month, 1‐year, and 2‐year cumulative incidence rate of first dose modification was 47%, 53% and 61%, respectively). Of these 43 patients, 17 patients increased the dose of ibrutinib, 9 patients decreased the dose, and the remaining 17 patients had at least one increase in dose and at least one decrease in dose. Reasons for dose modification (some dose modifications were due to multiple reasons) included: (a) nonhematologic toxicity (n = 19; atrial fibrillation [n = 2], bleeding [n = 5], diarrhea [n = 5], infection [n = 2], musculoskeletal pain [n = 3], other [n = 5]); (b) hematologic toxicity (n = 7); (c) drug‐drug interaction (n = 7); (d) physician/patient preference (n = 5); (e) other (n = 1).

Eighteen patients who started at reduced dose eventually received the standard dose (420 mg ibrutinib). Dose increases typically occurred after resolution of side effects (ie if patient had a previously decreased dose due to toxicity), after discontinuation of a concomitant drug that caused drug‐drug interaction, or at physician discretion based on tolerability at lower dose.

### 3.4 Dose interruption during ibrutinib therapy

During the 281 person‐years of treatment, 91/209 CLL patients held ibrutinib 142 times (estimated 1‐year and 2‐year rate of dose interruption was 40% and 57%, respectively). The primary reason for each dose hold included a) nonhematologic toxicity (n = 77, atrial fibrillation [n = 5], bleeding [n = 10], diarrhea [n = 9], infection [n = 15], musculoskeletal pain [n = 9], rash [n = 9], and other [n = 20]); b) hematologic toxicity (n = 14); c) drug‐drug interaction (n = 1); d) physician/patient preference (n = 2); e) procedures (n = 41); f) financial burden (n = 2) and g) other (n = 5). Of the 142 dose holds, 53 (37%) were for < 7 days, 50 (35%) for 7‐14 days, and 37 (26%) for ≥ 15 days; 2 could not yet be categorized at the time of analysis. Ibrutinib was held for a median of 13 days for procedures (range, 4‐55) and for 13 days for the 2 patients who were unable to get drug due to a change in their insurance policy. The primary reasons for holding ibrutinib stratified by the number of days that the medication was held are summarized in Table 2. There were no significant differences in the rates of ibrutinib interruption among patients who started standard versus reduced dose ibrutinib (*P* = .7). The estimated unadjusted 6‐month, 1‐year, and 2‐year risk of ibrutinib interruption (first event only) was 28%, 40%, and 57%, respectively. The median time to first dose interruption was 19 months.

**Table 2:** Primary reasons for temporary interruption in ibrutinib therapy for 140 holds (in 89 patients) categorized by length of interruption

Reasons for holding ibrutinib	<7 d N	7‐14 d N	≥15 d N
Procedure	7	21	12
Drug‐drug interaction	1	0	0
Hematologic toxicity	9	3	2
Non‐hematologic toxicity	34	21	21
Atrial fibrillation	2	1	2
Bleeding	2	5	2
Diarrhea	6	2	1
Infection	5	3	7
Musculoskeletal pain	6	3	0
Rash	2	4	3
Other****	11	3	6
Physician/patient preference	0	1	1
Financial Burden	0	2	0
Other****	2	2	1

*** Includes nausea, headache, dizziness, fever, fatigue, shortness of breath, blurred vision, night sweats, anosmia, hemolysis, pancreatitis, conjunctivitis, pruritus, hypercalcemia, worsening Raynauds.

**** Includes hospitalization, inability to swallow, trauma and patient preference.

### 3.5 Reasons for discontinuation of therapy

After a median time on ibrutinib therapy of 20 months, 61 patients discontinued ibrutinib; reasons for discontinuation of ibrutinib include: (a) toxicity (n = 37; atrial fibrillation [n = 3], infection [n = 8], bleeding [n = 4], cardiovascular event [n = 5], hematologic toxicity [n = 5], diarrhea [n = 3], musculoskeletal pain [n = 2], and other [n = 7]); (b) progression of CLL (n = 11); (c) progression due to Richter's transformation (n = 8); (d) financial burden (n = 2); and (e) patient preference (n = 3). The reasons for discontinuation differed according to the follow‐up interval. After 12, 24 and 36 months of follow‐up, an estimated 7%, 8%, and 20% of patients, respectively, discontinued due to progression of disease (both CLL progression and Richter's transformation) compared to 14%, 21%, and 28%, respectively, due to toxicity.

### 3.6 Outcomes

**Figure 1:**
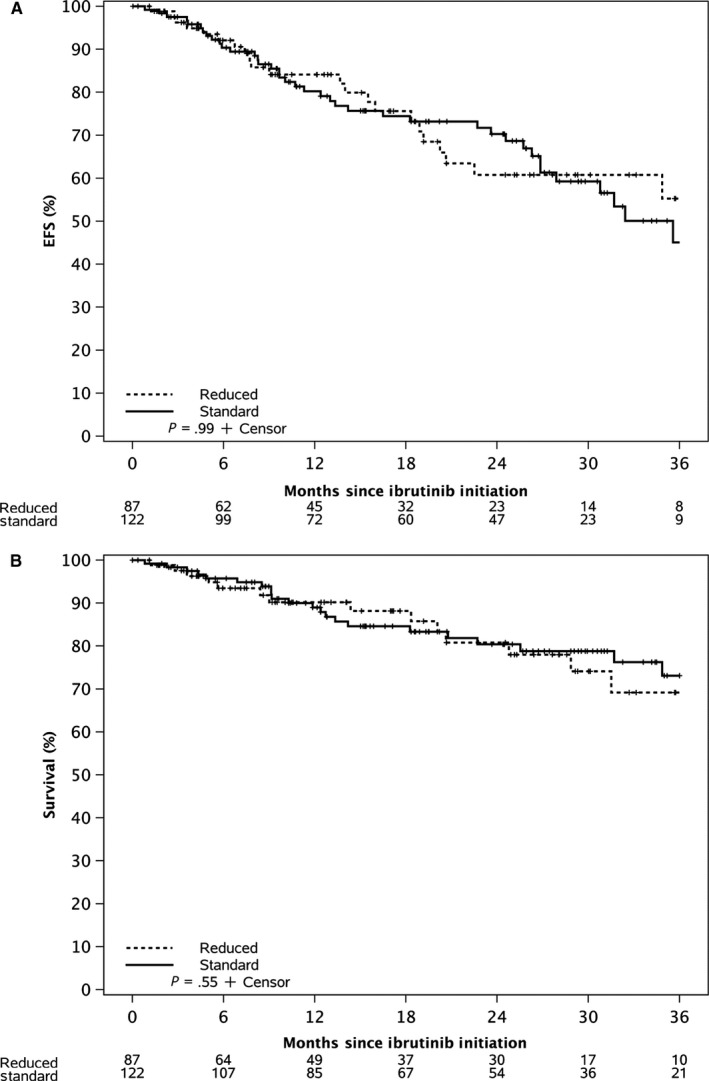
A, Event Free Survival of CLL patients who started therapy with standard dose ibrutinib (420 mg daily) compared to reduced dose ibrutinib (<420 mg daily) B, Overall Survival of CLL patients who started therapy with standard dose ibrutinib (420 mg daily) compared to reduced dose ibrutinib (<420 mg daily)

**Figure 2:**
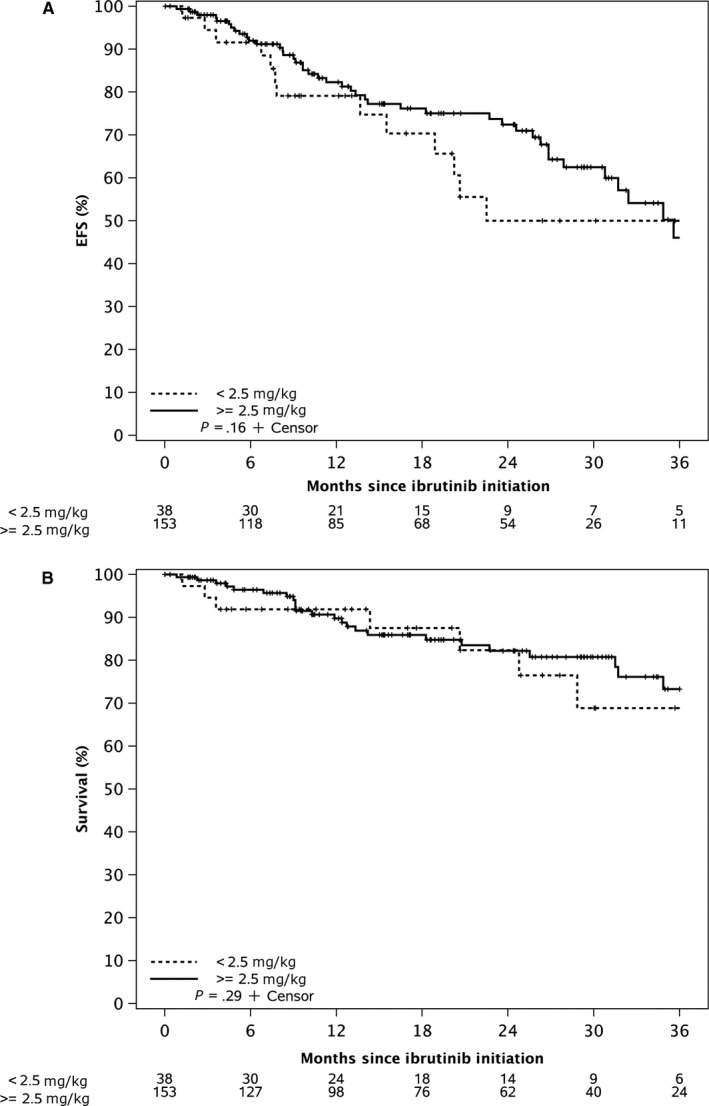
A, Event Free Survival of CLL patients who started therapy with ibrutinib at ≥2.5 mg/kg daily dose compared to <2.5 mg/kg daily dose. B, Overall Survival of CLL patients who started therapy with ibrutinib at ≥2.5 mg/kg daily dose compared to <2.5 mg/kg daily dose

The median follow‐up for all patients in the study is 24 months. The estimated median EFS for all patients from ibrutinib initiation was 36 months, and the median OS has not yet been reached. The EFS was not significantly different between patients who initiated standard dose ibrutinib compared to reduced dose ibrutinib (*P*‐value = .99, Figure 1A). OS was also not significantly different between these two groups (*P*‐value = .55, Figure 1B). After excluding 16 patients who were on concomitant medications that could decrease ibrutinib metabolism (such as CYP3A inhibitors), we found no statistically significant difference in the EFS and OS among patients who started ≥ 2.5 mg/kg/day ibrutinib versus those who received < 2.5 mg/kg/day (Figure 2A,B).

Table 3 shows the factors associated with EFS and OS in univariable analyses. After adjusting for age, sex, Rai stage, prior treatment status, and initial ibrutinib dose, temporary dose interruption of ibrutinib (hazard ratio [HR]: 2.37, 95% CI 1.29‐4.36, *P* = .006) and *TP53* disruption (HR: 1.81, 95% CI 1.01‐3.27, *P* = .048) were associated with a shorter EFS. In addition, after adjusting for age, prior treatment status, and initial ibrutinib dose, *TP53* disruption (HR: 2.38; 95% CI, 1.19‐4.76; *P* = .015) was associated with shorter OS however temporary dose interruption (HR: 1.98, 95% CI 0.98‐4.00, *P*‐value = .06) did not reach the threshold of statistical significance. Table S1 shows that patients who had a temporary dose interruption for nonhematologic toxicity were more likely to experience shorter EFS compared to patients who experienced temporary dose interruption for hematologic toxicity or surgical procedures, although there was no impact on OS. After adjusting for temporary dose interruptions, dose modification among patients who started standard dose ibrutinib was not associated with a shorter EFS (HR = 1.34, 95% CI 0.67‐2.66, *P* = .41) or shorter OS (HR = 2.11, 95% CI 0.87‐5.10, *P* = .10). In contrast, among patients who started reduced dose ibrutinib, and after adjusting for temporary dose interruptions, dose modification during the course of treatment was associated with shorter EFS (HR = 4.18, 95% CI 1.28‐13.70, *P* = .02) but not with shorter OS (HR = 2.19, 95% CI 0.67‐7.18, *P* = .20).

**Table 3:** Univariable factors associated with shorter event‐free survival (EFS) and overall survival (OS) among patients treated with ibrutinib

Characteristic	EFS		OS	
	HR (95% CI)	*P*‐value	HR (95% CI)	*P*‐value
Age at ibrutinib per 10 y increase	1.11 (0.87‐1.41)	.41	1.32 (0.97‐1.81)	.08
Female sex	1.37 (0.82‐2.29)	.22	1.66 (0.88‐3.14)	.12
Rai Stage (reference Rai 0)		.73		.40
Rai I/II	0.72 (0.32‐1.61)		0.55 (0.19‐1.64)	
Rai III/IV	0.84 (0.42‐1.70)		0.96 (0.39‐2.34)	
*IGHV* unmutated	1.03 (0.55‐1.93)	.92	0.84 (0.38‐1.90)	.68
High‐risk FISH (del11q and del17p)	0.98 (0.58‐1.66)	.94	1.15 (0.59‐2.24)	.68
*TP53* disruption (del17p or *TP53* mutation)	1.67 (0.96‐2.91)	.07	2.26 (1.14‐4.50)	**.02**
Reduced initial dose (<420 mg daily)	1.00 (0.61‐1.64)	.99	1.22 (0.64‐2.30)	.55
<2.5 mg/kg/d initial dose	1.50 (0.85‐2.67)	.17	1.50 (0.70‐3.21)	.29
Temporary dose interruption for any reason**	2.23 (1.27‐3.91)	**.005**	2.15 (1.08‐4.29)	**.03**

** Time dependent variable set up as time to first occurrence.

Bold indicates *P*‐value < .05

## 4 DISCUSSION

The introduction of BTK inhibitors such as ibrutinib has revolutionized the treatment landscape for patients with CLL. Although initial results from pivotal clinical trials demonstrated impressive responses, with significant improvement in PFS and OS compared to standard therapy,^1^, ^2^ more recent “real‐world” evidence suggest that there are important differences in dosing patterns, reasons for discontinuation and outcomes of patients treated with commercial supply of ibrutinib.^7^, ^13^ Results from our study show that among CLL patients treated outside the context of a clinical trial, approximately 40% patients initiated ibrutinib at a reduced dose (<420 mg daily, primarily due to concomitant medications that may increase ibrutinib toxicity and physician prescribing patterns), and approximately 50% patients subsequently undergo a dose modification and/or a dose interruption after initiation of ibrutinib therapy. Although ibrutinib starting dose and dose modifications did not impact EFS and OS, temporary dose interruptions during therapy were associated with shorter EFS and shorter OS.

Results from our study indicate that of the 209 patients who initiated ibrutinib in routine clinical practice, approximately 15% patients are on concomitant medications (such as CYP3A interacting medications or medications that can increase the risk of bleeding), that precluded the use of standard initial dose of ibrutinib. An additional 20% of patients initiate ibrutinib at reduced dose primarily due to the age or co‐morbidities that, in the opinion of the prescribing provider, precluded the use of standard dose ibrutinib. In the original phase 1 study of ibrutinib with relapsed B‐cell malignancies (including CLL, mantle cell lymphoma and follicular lymphoma, diffuse large B‐cell lymphoma and marginal zone lymphoma); the maximum tolerated dose of ibrutinib was not reached. After oral absorption, peak plasma concentrations of ibrutinib were achieved in 1‐2 hours, and drug exposure, as measured by area under the concentration‐time curve, increased in a dose proportional manner. Measurement of BTK occupancy at multiple time points indicated sustained occupancy (>95%) at all dose levels where the AUC exceeded 160 ng**·**h/mL, which corresponded to ≥2.5 mg/kg/day dose of ibrutinib.^14^ In our series, no differences in EFS or OS were observed based on whether the starting dose of ibrutinib was above or below 2.5 mg/kg/day. Recent data from MD Anderson Cancer Center also suggest that patients who initiate ibrutinib at lower dose (420 mg daily for 4 weeks, followed by 280 mg daily for 4 weeks, and 140 mg daily for 4 weeks) may have similar outcomes to patients who are on standard dose ibrutinib.^15^ Whether lower doses of ibrutinib may be equally effective and less toxic needs to be investigated in a properly conducted clinical trial, and such a strategy is being planned.^15^ Although a lower starting dose in a subset of patients did not seem to impact outcomes in our patients, longer term follow‐up in larger cohorts of patients (preferably done in the context of a clinical trial) are necessary before this may be considered as standard practice. Therefore, at the current time, we recommend all patients receive therapy according to the manufacturer's prescribing information. Our results also show that dose modifications are a relatively common occurrence during ibrutinib therapy—occurring in approximately 50% patients in our study after 2 years of follow‐up. The most common reasons for reducing the dose of ibrutinib in our cohort was nonhematologic toxicity, with atrial fibrillation, bleeding and diarrhea being the most common nonhematologic toxicities prompting a dose change. Such clinically indicated dose reductions had no apparent impact EFS and OS. Dose interruptions were also fairly common in routine clinical practice, with an estimated 60% patients requiring dose interruptions at 2 years. Notably, temporary dose interruptions were associated with shorter EFS and OS, suggesting that patients who are able to adhere to treatment better may derive more benefit. These results complement data from Barr and colleagues where CLL patients treated on the RESONATE trial who demonstrated sustained adherence to ibrutinib therapy (examined during the first 8 weeks on therapy) had an improved progression‐free survival and OS.^5^ Findings from our study extend these observations to include adherence to therapy not only during the first 8 weeks, but during the entire course of therapy may impact outcomes. These findings should be interpreted with caution since patients who are able to adhere to therapy are generally those who are able to tolerate treatment better, and have no major comorbidities. In this regard, patients who held ibrutinib for surgical procedures had better EFS and OS compared to those patients who held ibrutinib for toxicity.

Similar to the findings of Mato and colleagues, our study also found that more patients stopped ibrutinib due to toxicity compared to progression of disease. This underscores the importance of combining ibrutinib with other agents that will allow stopping therapy to mitigate toxicity, in contrast to indefinite use as is current practice. Although the addition of rituximab to ibrutinib did not show any benefit,^16^, ^17^ the ILLUMINATE study (combining ibrutinib and obinutuzumab) showed that ~35% patients achieved minimal residual disease negative complete remission.^18^ The CLL14 trial (using a combination of venetoclax and obinutuzumab for fixed duration of 1 year),^19^ and a phase 2 study from MD Anderson Cancer Center (using a combination of ibrutinib and venetoclax for a fixed duration of 2 years)^20^ in frontline CLL show very high rates of minimal residual disease negative remission (>60%‐70%), highlighting that indefinite use of ibrutinib may not be necessary in all patients.

Our study has several limitations. First, it is a single‐center retrospective study with a short follow‐up of <3 years, and the findings may not be generalizable. Second, during the study interval (2013‐2017), the understanding of ibrutinib‐related toxicities (such as whether or not safe to combine with anticoagulation), and other aspects of management of patients on ibrutinib evolved. Finally, other novel agents such as idelalisib and venetoclax were approved by the FDA during the conduct of this study, providing physicians more choices of therapy of relapsed/refractory CLL, which may have altered practice patterns (particularly with respect to stopping treatment with ibrutinib for toxicity due to availability of alternate effective therapy).

In summary, in a large cohort of CLL patients receiving commercial ibrutinib, ~40% patients started at a lower dose of ibrutinib. Despite this, the starting dose (analyzed both by fixed dose or weight based dosing) of ibrutinib, and dose modifications during the course of therapy did not impact outcomes, whereas temporary dose interruption for any reason appeared to be associated with shorter EFS and OS. Our results also confirm observations from other “real‐world” analyses that more patients stop ibrutinib due to toxicity than due to progression of disease. This latter finding has major implications for clinical trial design where combination therapies with fixed duration of treatment that achieve deep remission may be more desirable than chronic long‐term treatment.

## CONFLICTS OF INTEREST

SAP: Research funding has been provided to the institution from Pharmacyclics, MorphoSys, Janssen, AbbVie, AstraZeneca, Genentech, GlaxoSmithKline, Celgene, and Ascentage Pharma for which Sameer A. Parikh is an investigator. He has also participated in Advisory Board meetings of Pharmacyclics, AstraZeneca, Gilead, and AbbVie (he was not personally compensated for his participation). NEK: Research funding has been provided to the institution from Pharmacyclics, Acerta, Tolero & MEI Pharma for which Neil E. Kay is a principal investigator. Dr Kay has also participated as a member of the Data and Safety Monitoring Board for Cytomx Therapeutics, Infinity Pharm, Agios Pharm, Celgene, and MorphoSys. He has also participated in Advisory Board meetings of Pharmacyclics and Janssen. WD: Research funding has been provided to the institution from Merck for which Wei Ding is a principal investigator. SSK: Sponsored Research Funding provided to the laboratory from Novartis, Kite, Actinium, Morphosys, Lentigen, Humanigen and Tolero. SSK is inventor on patents licensed to Novartis and Humanigen. SSK is a co‐founder of LeahLabs and SensImmune and he has participated as a consultant to Leahlabs and Kiniska. TDS: Research funding has been provided to the institution from Pharmacyclics, Janssen, Genentech, Glaxo‐SmithKline, Celgene, Cephalon, and Hospira for which Tait D. Shanafelt is a principal investigator.

## AUTHOR CONTRIBUTION

SAP, HDF, and TDS designed the research, collected, analyzed and interpreted data, cared for the patients and wrote the manuscript. TGC, WD, JFL, SSK, AAC, ABK, EM, ALF, KBM, ANN, and MDS cared for the patients, analyzed data, and critically reviewed the manuscript. SJA, KGR, SMS, and SLS collected and analyzed data, conducted statistical analysis, and critically reviewed the manuscript. All authors approved the manuscript in its final format.

## Supporting information

Table S1

## Data Availability

Research data are not shared.
