# Supplementary material for: The impact of dose modification and temporary interruption of ibrutinib on outcomes of chronic lymphocytic leukemia patients in routine clinical practice
Source: Cancer Med. 2020 Mar 18;9(10):3390–9. doi: 10.1002/cam4.2998 (PMC7221301; doi:10.1002/cam4.2998)
Supplement: Supplementary file 1 — Table S1 [file CAM4-9-3390-s001.docx]

**Supplemental Table 1**: Univariable analyses of specific reasons for dose interruption** with shorter event free survival and overall survival

| Characteristic | EFS | | OS | |
| --- | --- | --- | --- | --- |
|  | HR (95% C.I.) | p-value | HR (95% C.I.) | p-value |
| Temporary dose interruption for any reason | 2.23 (1.27-3.91) | **0.005** | 2.15 (1.08-4.29) | **0.03** |
| Temporary dose interruption for non-hematologic toxicity | 2.23 (1.33-3.74) | **0.002** | 1.65 (0.83-3.29) | 0.15 |
| Temporary dose interruption for hematologic toxicity | 1.20 (0.38-3.80) | 0.76 | 1.69 (0.53-5.34) | 0.37 |
| Temporary dose interruption for procedure | 1.06 (0.48-2.34) | 0.88 | 1.31 (0.51-3.39) | 0.58 |

**time dependent variable
